# In-silico investigations of haemodynamic parameters for a blunt thoracic aortic injury case

**DOI:** 10.1038/s41598-023-35585-8

**Published:** 2023-05-23

**Authors:** Rezvan Dadras, Alireza Jabbari, Narges Kamaei Asl, Madjid Soltani, Farnaz Rafiee, Mozhgan Parsaee, Shadi Golchin, Hamidreza Pouraliakbar, Parham Sadeghipour, Mona Alimohammadi

**Affiliations:** 1 Department of Mechanical Engineering, K. N. Toosi Univeristy of Technology, Tehran, Iran; 2 Rajaie Cardiovascular, Medical, and Research Center, Iran University of Medical Sciences, Tehran, Iran

**Keywords:** Biomedical engineering, Cardiovascular diseases, Computational models, Translational research

## Abstract

Accounting for 1.5% of thoracic trauma, blunt thoracic aortic injury (BTAI) is a rare disease with a high mortality rate that nowadays is treated mostly via thoracic endovascular aortic repair (TEVAR). Personalised computational models based on fluid–solid interaction (FSI) principals not only support clinical researchers in studying virtual therapy response, but also are capable of predicting eventual outcomes. The present work studies the variation of key haemodynamic parameters in a clinical case of BTAI after successful TEVAR, using a two-way FSI model. The three-dimensional (3D) patient-specific geometries of the patient were coupled with three-element Windkessel model for both prior and post intervention cases, forcing a correct prediction of blood flow over each section. Results showed significant improvement in velocity and pressure distribution after stenting. High oscillatory, low magnitude shear (HOLMES) regions require careful examination in future follow-ups, since thrombus formation was confirmed in some previously clinically reported cases of BTAI treated with TEVAR. The strength of swirling flows along aorta was also damped after stent deployment. Highlighting the importance of haemodynamic parameters in case-specific therapies. In future studies, compromising motion of aortic wall due to excessive cost of FSI simulations can be considered and should be based on the objectives of studies to achieve a more clinical-friendly patient-specific CFD model

## Introduction

Among all trauma-related fatality, thoracic trauma is the reason of approximately 25% of deaths^1^. Accounting for 1.5% of thoracic trauma, blunt thoracic aortic injury (BTAI) is a rare and mostly fatal disease with in-hospital mortality rate of 46%^2^. BTAI is a tear caused by a combination of shear forces and increased intravascular pressure wherein rapid deceleration exists in most cases^3^. Motor vehicle collision is the foremost common cause of BTAI with 81% of cases^4^. Injury is possible anywhere along the aorta; from ascending aorta to iliac bifurcation notably aortic isthmus^5^. Currently the classification of BTAI is based on the involvement of aortic wall layers; Grade I (intimal tear), Grade II (intramural hematoma), Grade III (pseudo aneurysm), and Grade IV (rupture)^6,7^. In majority of BTAI cases (50.3%) a pseudo aneurysm is present (grade III) wherein intima, media, and adventitia layers are torn^8^. This pseudo aneurysm creates local changes in the area of the lumen and is prone to creation of narrowing regions. The locally narrowed areas which are also observed in patients suffering from coarctation, are seen to cause swirling flows, proximal and distal to the narrowed region specially during deceleration and diastolic phase of cardiac cycle. Furthermore, these disorganised flows result in twisting and instability of their downstream^9–12^ and are observed to result in swirling patterns of endothelial cells which are suggestive of vortices several millimeters downstream from the narrowed region^13^. Blood acceleration and complex downstream recirculation across these regions leads to a jet impinging on the aortic wall^14^ which may further lead to aortic wall dilation^11,15^ and even rupture^15^.

The recommended standard treatment in adults suffering from BTAI, is thoracic endovascular aortic repair (TEVAR)^17^. Seemingly thoracic endovascular grafts are safe and effective for the treatment of BTAI, for both short-term and follow-up results. These devices lead to very low rates of aortic injury-related mortality^17–19^. However, serious attention is required in further follow-ups for immediate diagnosis of consequent problems. In a recent study, on long–term outcomes of using these grafts in patients suffering from BTAI, late formation of thrombus; most commonly at the distal aspect of the stent graft was confirmed in 26% of the patients after TEVAR^17^. Also, iatrogenic coarctation as a result of device underexpansion and aortic remodeling has also been reported which predispose patients to long-term consequences^20^. Therefore, studying therapy response by comparing patients’ condition before and after the operation, helps clinical researchers to identify possible follow-up problems as well as creating systematic framework in treatment of BTAI.

In recent years a number of studies have been done on BTAI, focusing on simulating the motor vehicle accident, its impact on thoracic region, and possibility of blunt thoracic aortic rupture (BTAR). For instance, Di Labbio et al.^21^ studied the evolution of pulsatile flow in a circular cross-sectional pipe subjected to a transverse impulse body force and declared that although the corresponding transverse wall shear stress may not actually cause rupture, it probably has significant role in occurrence of rupture away from aortic isthmus. Wei et al.^22^ used a model based on finite element methods (FEM) for the aorta-heart system and developed a real car crash scenario by considering both hydrodynamic and kinematic effects. The study tried to predict aortic isthmus laceration and other possible injuries on ribs and diaphragm during motor vehicle collision. In agreement with previous study others showed that max stress occurs in the isthmus region. It was also concluded that comparing the stretching that occurs at the ascending aorta to the bending generated at the aortic arch, bending is a leading factor in BTAR^23,24^.

Personalised computational fluid dynamics (CFD) along with FEM, with the ability to represent haemodynamic metrics, can support physicians in terms of management of aortic diseases, predicting possible future scenarios, and leading to design and analyse of new therapy methods^25–28^. In addition, CFD tools coupled with machine learning algorithms have promising future potential to increase the accuracy of simulations, as well as reducing the computational costs^29^. However, despite the potentials of numerical simulations, to the best of author’s knowledge, no single study has been reported to utilise CFD analysis based on fluid–solid interaction (FSI) principals, in a clinical case of BTAI to consider the variation of haemodynamic parameters before and after the treatment; therefore, the prior purpose of this study is to demonstrate the efficacy of treatment on a patient who had suffered from BTAI and also to prognosticate possible long-term outcomes of stenting, using FSI modelling along with available patient-specific data.

In this study two patient-specific cases were driven from pre and post intervention aortic computed tomography angiography (CTA) images of a 30-year–old male suffering from BTAI considering inclusion of aortic wall motion. For each case, personalised geometry of the patient was extracted from CTA images and was coupled with tuned three element Windkessel model. The velocity profile of the patient was also integrated using pulse wave Doppler echocardiography. Finally, to comprehend the treatment outcomes, following parameters were calculated and compared in both prior and post intervention phases;

1. Velocity and streamline distribution
2. Pressure distribution
3. Wall shear stress (WSS) indices
4. Swirling strength (SS)
5. Aortic wall displacement
6. Von Mises stress

What is more, by compromising aortic wall motions to reduce the cost of simulations and therefore, using the basics of this model in future personalised simulations, various invasive, non-invasive, or combination of both treatments can be applied on prior to intervention case virtually before any medical or surgical procedures. This eventually helps clinical researchers obtain the best case-specific solution for BTAI allowing better treatment planning for such patients.

## Results

### Velocity and streamline distribution

**Figure 1:**
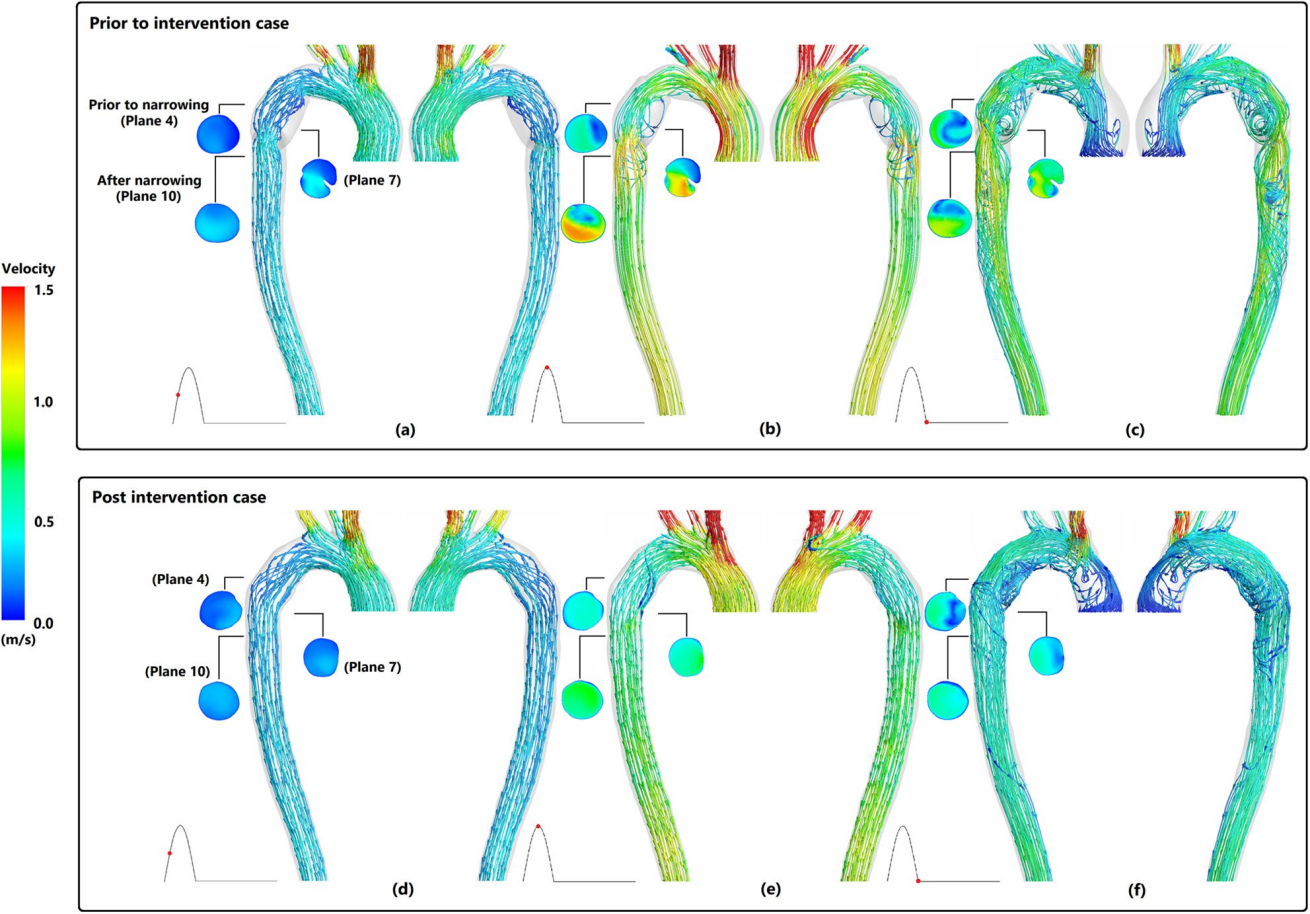
Velocity and streamline distribution on right anterior and left posterior for prior and post intervention case. (**a**,**d**) mid systole, (**b**,**e**) peak systole, and (**c**,**d**) diastole.

Figure 1 shows the forward and backward streamline patterns of both prior and post intervention for three major stages of cardiac cycle. In both cases; during mid systole (Fig. 1a,d) uniform streamlines are observed along ascending aorta (AA) and aortic arch prior to pseudo aneurysm Some mild vortices through pseudo aneurysm in prior intervention case can be seen. Figure 1b,e illustrate moderate vortices along and after the narrowing region of pseudo aneurysm. This is due to increase of flow velocity during peak systole in prior intervention, which are not present in post intervention case. Although both cases demonstrate disordered streamlines with chaotic vortices in aortic arch during diastole (Fig. 1c,f); in prior intervention case high-velocity swirling flow due to local narrowing is observed along descending aorta (DA). The velocity contour of three selected cross-sectional planes for both cases are also presented in Fig. 1. The high value of velocity seems to be damped in all post intervention cases and the difference between maximum and minimum values of velocity has been reduced after stent deployment, resulting in uniformity of velocity fields over each cross-section. What is more, prior to intervention, brachiocephalic trunk (BT), left common carotid artery (LCC), and left subclavian artery (LS) received 31.66%, 9.60%, and 15.80% of the total inlet flow during systolic phase of cardiac cycle respectively, as opposed to 26.30%, 10.73%, and 18.20% after TEVAR (Table 1) The resulting 16.93% of decrease in BT flow rate after removing the coarcted region via treatment, boosted blood distribution to LCC and LS and also led to 6.55% of flow increase for DA.

**Table 1:** Percentage of flow distribution for both prior and post intervention cases during systolic phase.

	BT (%)	LCC (%)	LS (%)	DA (%)
Prior to intervention	31.66	9.60	15.80	41.23
Post intervention	26.30	10.37	18.20	43.93

**Figure 2:**
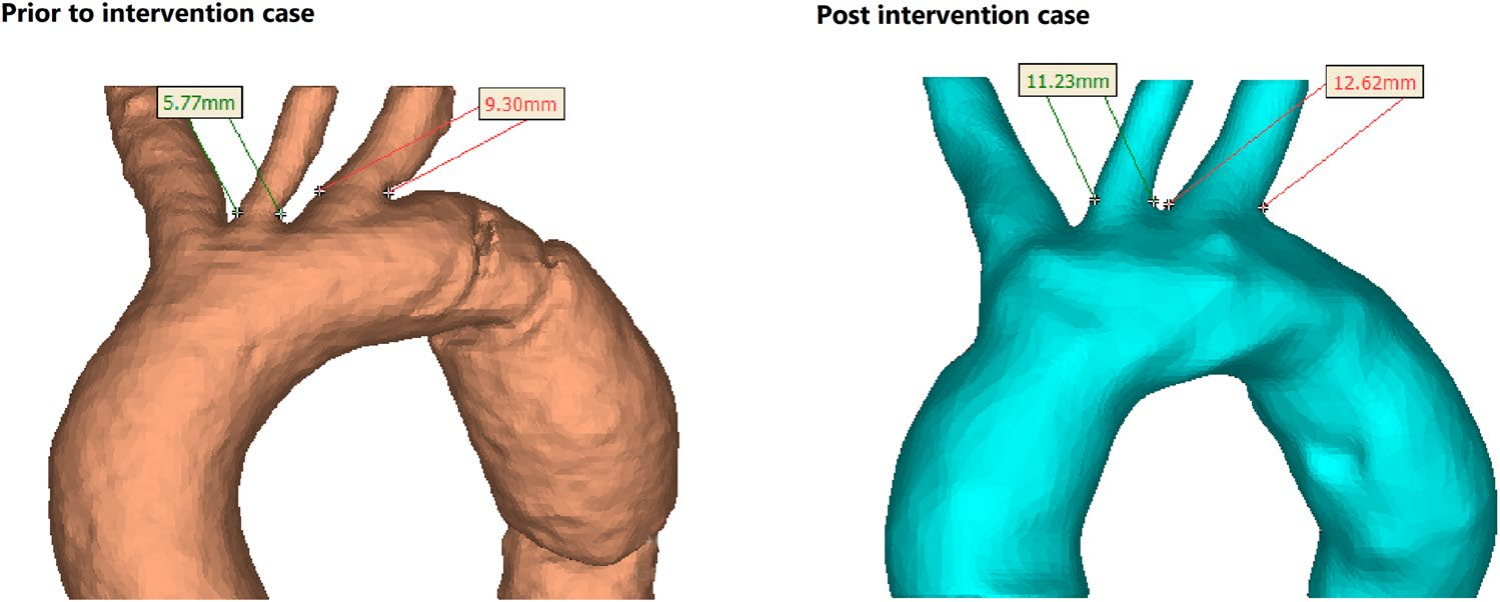
Geometry change in aorta after stenting.

Figure 2 shows upper branches more closely for both cases. It can be observed that before intervention due to special geometry of the patient’s aorta, LCC and LS have narrower roots with smaller cross section areas that can lead to higher velocity magnitudes. This is in agreement with lower velocity magnitudes in LCC and LS after stent deployment (Fig. 1).

### Pressure distribution

**Figure 3:**
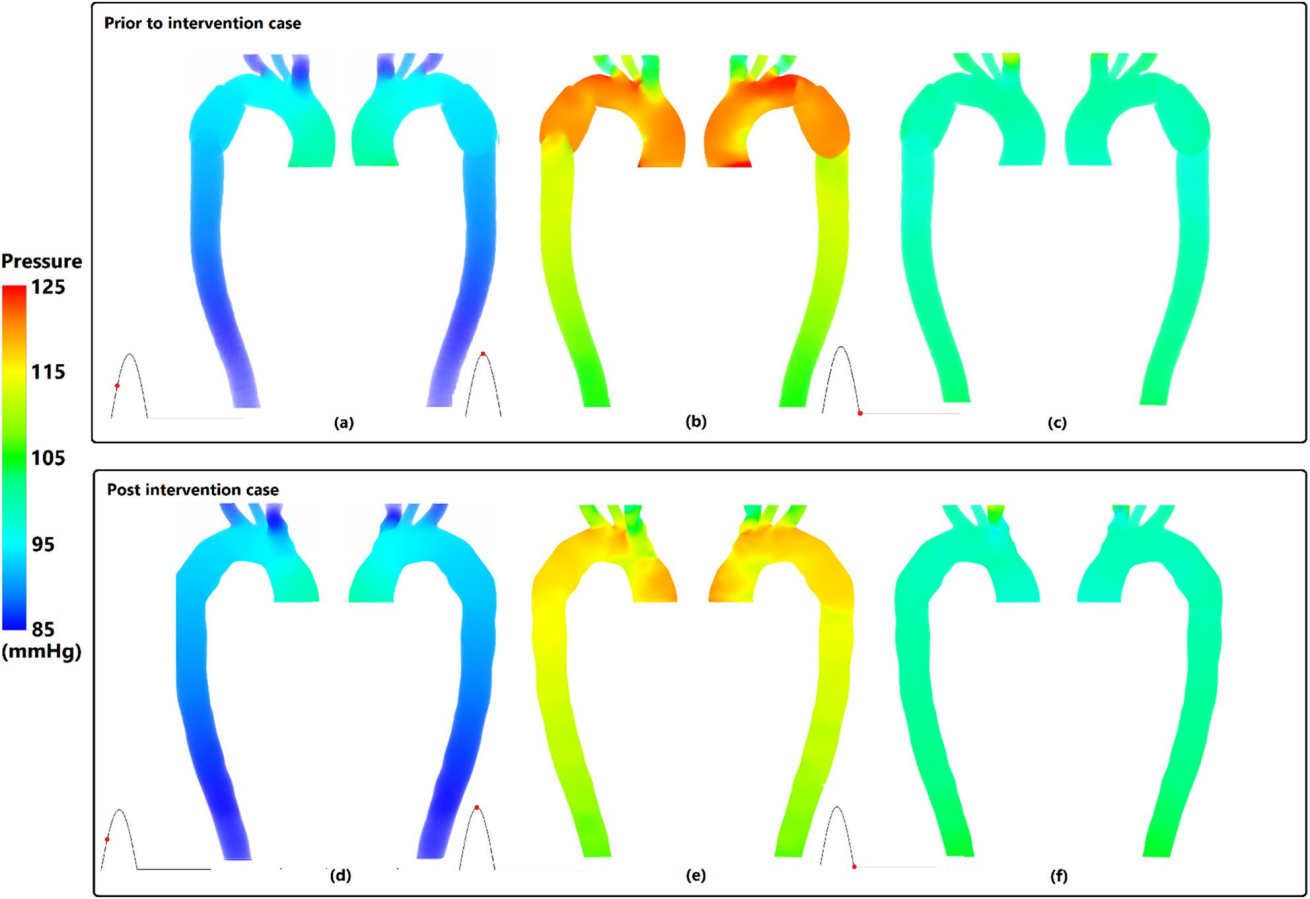
Pressure distribution on right anterior and left posterior for prior and post intervention cases. (**a**,**d**) mid systole, (**b**,**e**) peak systole, and (**c**,**d**) diastole.

The pressure distribution along the interested region of affected aorta is shown in Fig. 3 during three major time points of cardiac cycle for both cases. The highest pressure is observed at both mid and peak systole along AA (Fig. 3a,b,d,e) as opposed to the diastole point where the entire aorta shows a relative similar pressure (Fig. 3c,f); about 105 mmHg. Additionally, during mid systole and peak systole, the pressure drop is shown to be the same but with different absolute values. At peak systole (Fig. 3b,e), a sudden pressure decrease can be observed at aortic arch. However, the value of pressure has decreased further for the post intervention case; highlighting the efficacy of the existence of stent.

### Wall shear stress (WSS) indices

**Figure 4:**
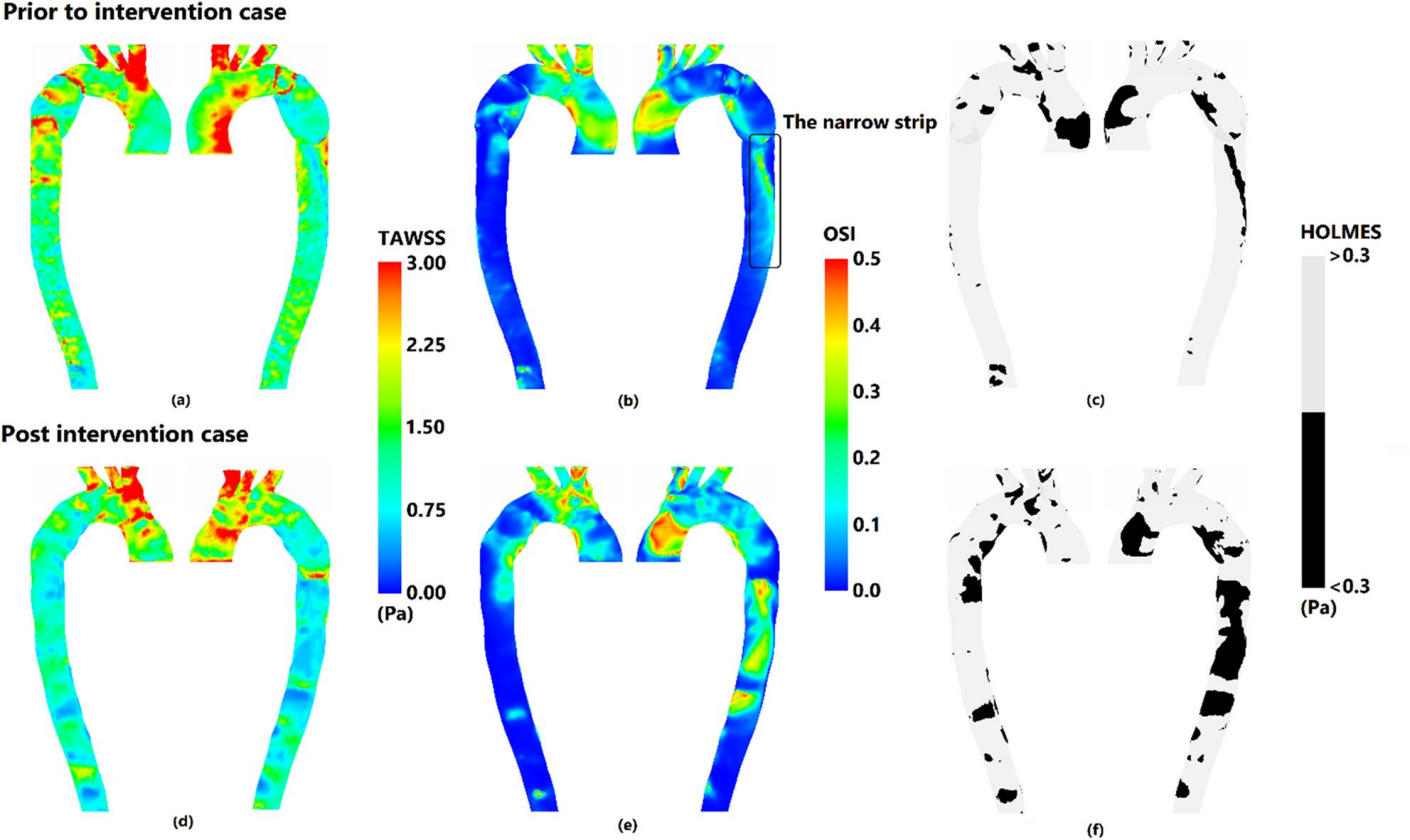
Wall shear stress indices on right anterior and left posterior for prior and post intervention cases. (**a**,**d**) TAWSS (Time average wall shear stress), (**b**,**e**) OSI (Oscillatory shear index), and (**c**,**d**) HOLMES (High oscillatory low magnitude shear index).

Performing patient-specific simulations, several WSS indices are commonly used to delineate the distribution of WSS as meaningful spatial parameters^31^. Based on the following equation^32^, the time average of WSS (TAWSS):

1 $$\mathrm{TAWSS}=\frac{1}{\mathrm{T}}{\int }_{0}^{\mathrm{T}} |\overrightarrow{\mathrm{wss}}|\mathrm{dt}$$

wherein $T$ is the length of cardiac cycle $(0.731 s)$ and $\overrightarrow{wss}$ is the magnitude of wall shear vector is derived on the vessel wall for both cases and is shown in Fig. 4a,d. Intensive and scattered high values of TAWSS due to specific curvature of aorta are observed in anterior AA for both prior and post intervention cases respectively. In addition, aortic arch prior to pseudo aneurysm, posterior pseudo aneurysm, and the local narrowing of prior intervention hold high values of TAWSS comparing prior to post intervention case. Reduction of moderate values of TAWSS along DA is also observed in post intervention in contrast to prior intervention case.

Oscillatory shear index (OSI) is a dimensionless WSS index, which shows the effect of oscillatory forces on endothelial cells^31^. This parameter has a value between 0 and 0.5 and is defined as:

2 $$\mathrm{OSI}=0.5\left(1-\frac{\left|\frac{1}{\mathrm{T}}{\int }_{0}^{\mathrm{T}} \overrightarrow{\mathrm{wss}}\mathrm{ dt}\right|}{\mathrm{TAWSS}}\right)$$

High values of OSI located in posterior AA of prior to stenting case are not present in post intervention as opposed to high OSI area in the anterior AA, that have been expanded after stenting (Fig. 4b,e).

The narrow strip of moderate values of OSI that is found in anterior pseudo aneurysm and continues along DA in prior to intervention case, confronts considerable enlargement of area and magnitude after stenting. In addition, looking into the same figure aortic arch faced higher magnitude of OSI after stent deployment.

To compare prior and post intervention case, regions of low-magnitude TAWSS and high OSI are of high importance. High oscillatory, low magnitude shear (HOLMES) (Eq. 3) as a modified form of TAWSS, indicates these regions^33^. Low and high magnitudes of HOLMES are presented in Fig. 4c,f for both cases. It can be seen that regions of low magnitude HOLMES follows the approximate pattern of high magnitude OSI and low TAWSS.

3 $$\mathrm{HOLMES}=\mathrm{TAWSS}\times (0.5-\mathrm{OSI})$$

### Swirling strength (SS)

**Figure 5:**
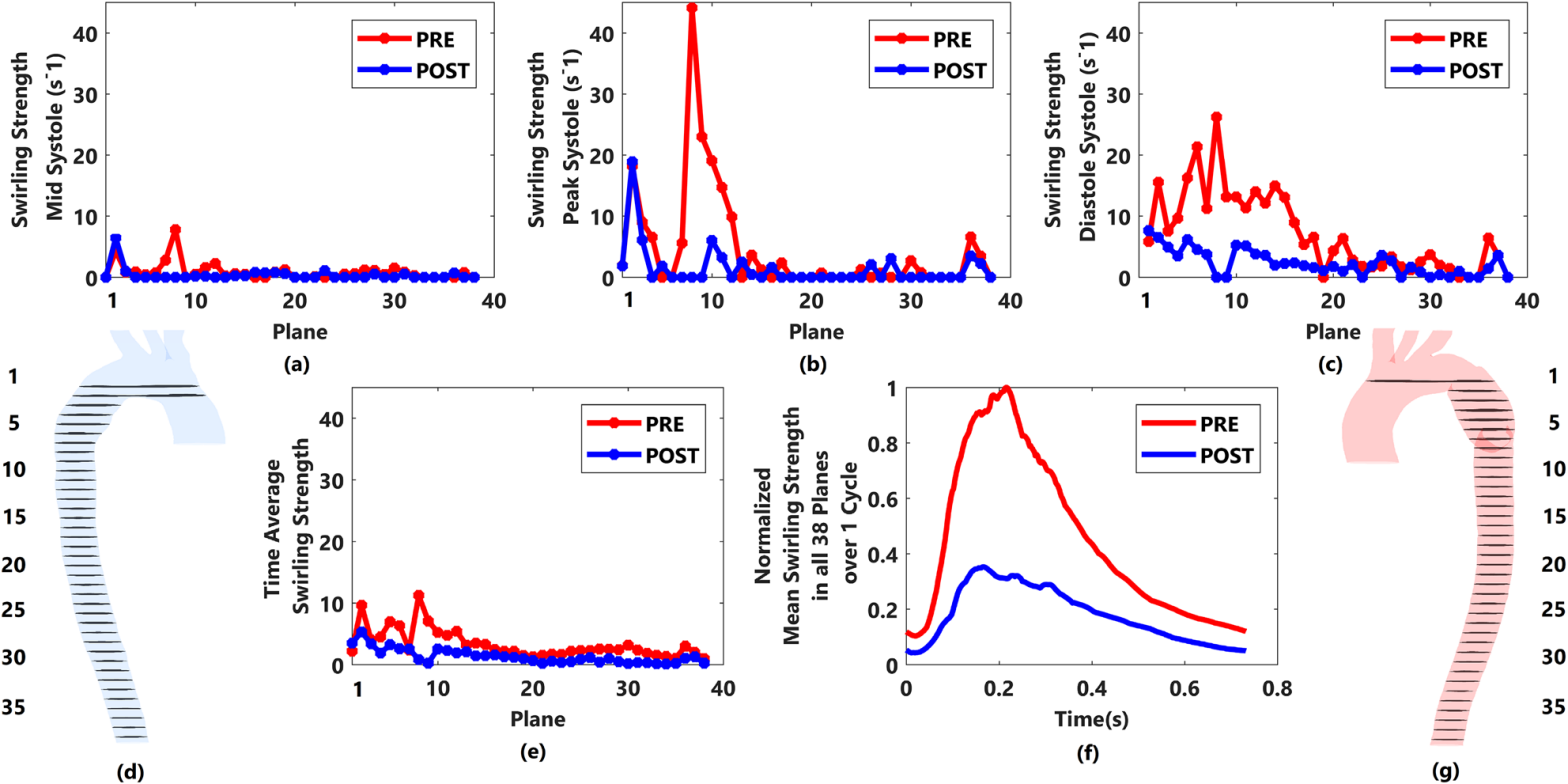
Swirling strength and plane locations. (**a**) SS (swirling strength) along planes for both cases during mid systole, (**b**) SS (swirling strength) along planes for both cases during peak systole, (**c**) SS (swirling strength) along planes for both cases during diastole, (**d**,**g**) Plane locations for both cases, (**e**) TASS (time average swirling strength) along planes for both cases, and (**f**) normalised mean SS (swirling strength) in all 38 planes during one cardiac cycle.

Figure 5 shows the SS of blood through all regions at different cardiac time points. The swirling strength shows the strength of the blood as it passes each region toward downstream vessels. This parameter is introduced by Zhou et al.^34^ and Adrian et al.^35^ and has been validated by Qi-Qiang^36^, that greater absolute value of SS, results in stronger internal circulation. SS is defined as the imaginary part of the complex Eigen value of the velocity gradient tensor $(\overrightarrow{\overrightarrow{J}})$, wherein $U, V,$ and $W$ are velocity components in $x, y,$ and $z$ direction respectively;

4 $$\overrightarrow{\overrightarrow{J}}=\nabla \overrightarrow{U}=\left[\begin{array}{lll}\partial U/\partial x& \partial U/\partial y& \partial U/\partial z\\ \partial V/\partial x& \partial V/\partial y& \partial V/\partial z\\ \partial W/\partial x& \partial W/\partial y& \partial W/\partial z\end{array}\right]$$

Thirty-eight planes were created along the aorta for the SS calculation in both cases (Fig. 5d,g) as one cannot calculate this for the entire volume of aortae. Thus, the insertion of more planes results in better approximation of SS. Plane one, is located prior to the pseudo aneurysm region, the pseudo aneurysm continues from plane one to seven, and from plane eight onwards (eight to thirty-eight) are inserted along DA.

During all three major time points (Fig. 5a–c), it can be seen that the overall SS for prior intervention case is greater than post intervention. As the cardiac cycle completes, the value of SS varies along the affirmation planes. However, proceeding the cardiac cycle, the upstream planes show higher values of SS relative to the downstream planes specifically for prior intervention case.

Time average swirling strength (TASS) is also introduced and has been derived in all planes to study both cases considering the changes of velocity gradient during one cardiac cycle. Figure 5e demonstrates the reduction of TASS after stenting. It can be seen that the eighth plane which is located immediately after the end of the local narrowing of pseudo aneurysm has the highest value of SS during all three major time points and holds the highest value of TASS to compare the general circulation of blood flow along the region of interest during one cardiac cycle, independent of the location of planes, the mean SS in all planes are found by adding up the values of SS for each plane and dividing the sum by the number of planes for both prior and post intervention cases. To remove any possible outliers, the normalised mean SS in all planes is presented in Fig. 5f. Being in agreement with other forms of SS, the normalised mean SS in all planes shows overall reduction of flow circulation

### Aortic wall displacement

**Figure 6:**
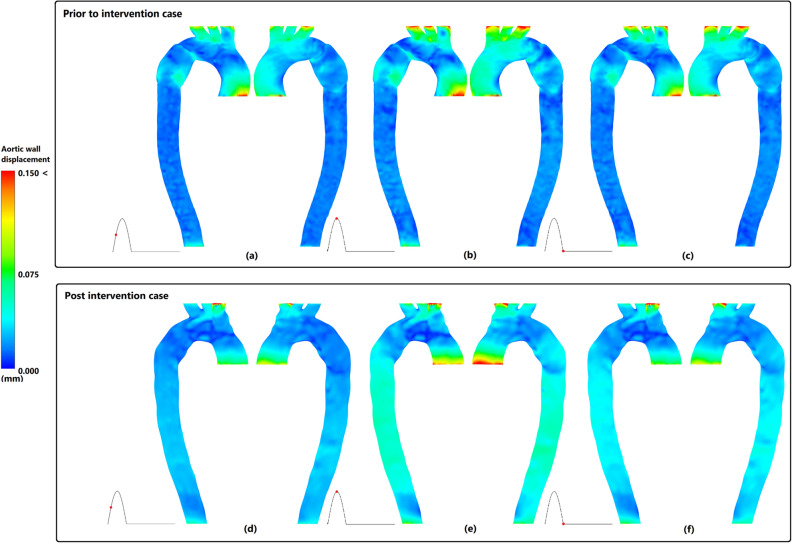
Aortic wall displacement in right anterior and left posterior for prior and post intervention cases. (**a**,**d**) mid systole, (**b**,**e**) peak systole, and (**c**,**d**) diastole.

Figure 6 indicates aortic wall displacement during three major time points of cardiac cycle for both prior and post intervention cases. During peak systole (Fig. 6b,e), AA and upper branches have the highest value of displacement in both cases. However, displacement of upper branches has decreased post stenting with increase in wall displacement along DA. The same pattern also exists in mid (Fig. 6a,d) and diastolic (Fig. 6c,f) phases with lower magnitudes.

### Von Mises stress

**Figure 7:**
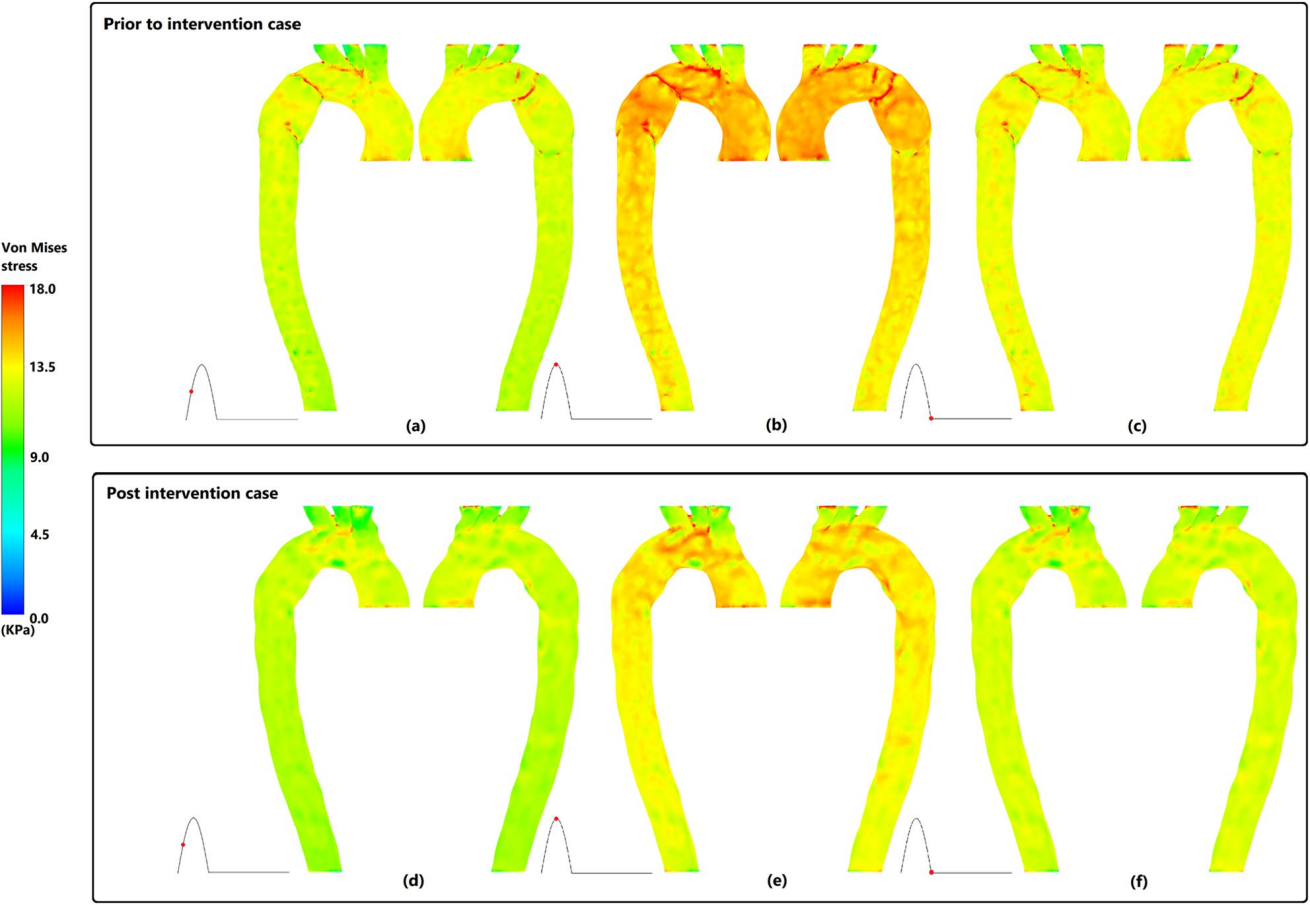
Von Mises stress on aortic wall in right anterior and left posterior for prior and post intervention cases. (**a**,**d**) Mid systole, (**b**,**e**) peak systole, and (**c**,**d**) diastole.

A standard method of predicting material destruction of aortic wall under haemodynamic forces is the measurement of Von Mises stress^37^. During mid systolic phase of prior to intervention case (Fig. 7a), high values of Von Mises stress are observed prior, along, and immediately after the end of pseudoaneurysm, root of the upper branches, and inner curvature of AA. The same pattern with greater magnitudes is seen during the peak and diastolic phases (Fig. 7b,c). Post stenting (Fig. 7d–f) root of the upper branches and inner curvature of AA still hold high values of Von Mises. However, overall reduction of Von Mises values along aortic wall with corresponding range of 3.78–29.44 kPa during peak systole is observed which is in contrast to 3.07–109.58 kPa for prior to intervention case.

### Effect of wall motion

**Figure 8:**
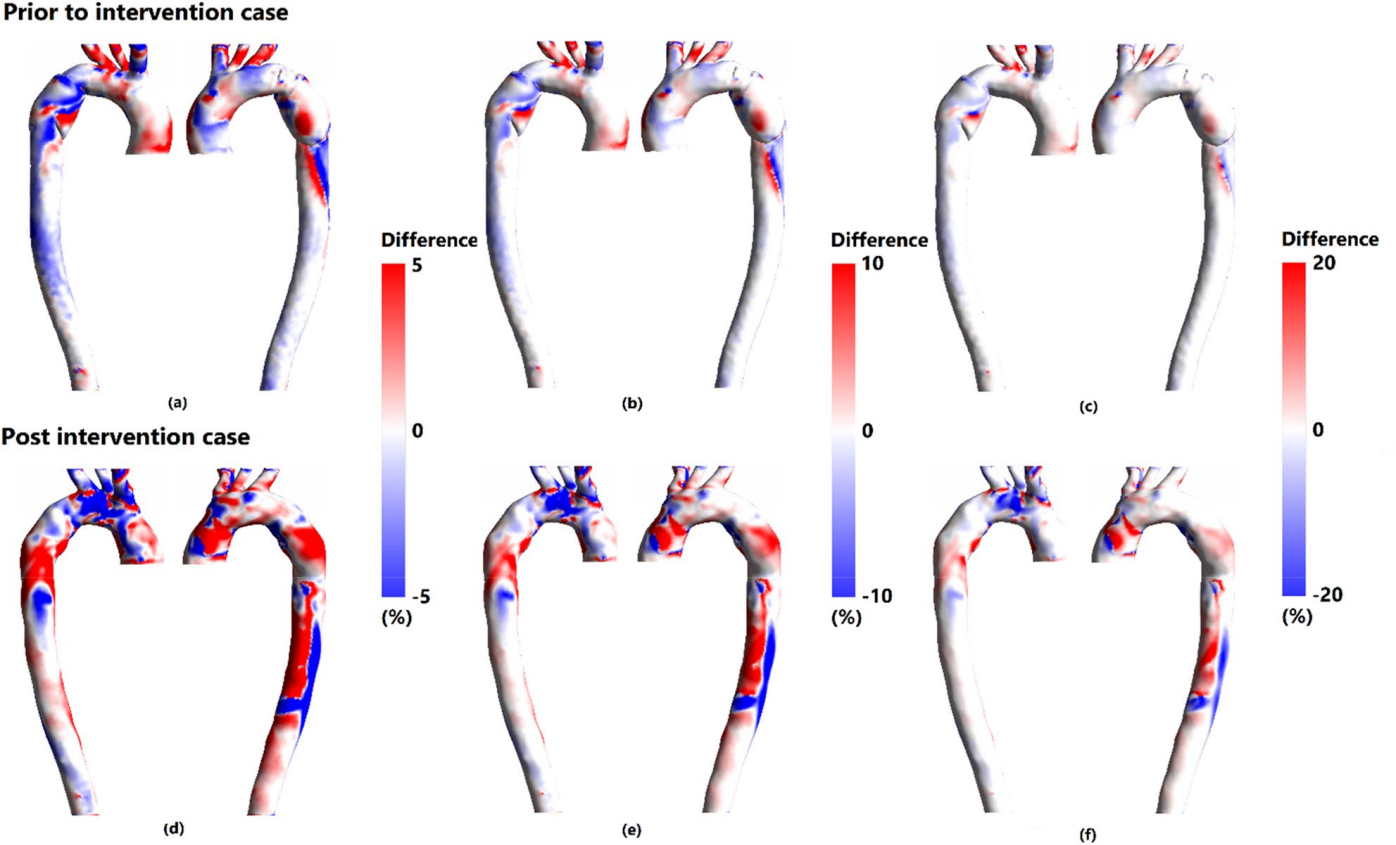
Percentage of HOLMES (Highly oscillatory, low magnitude shear) difference relative to rigid wall simulation for both cases using three color scales. (**a**,**d**) Percentage difference with absolute range of 5%, (**b**,**e**) Percentage difference with absolute range of 10% and, (**c**,**f**) percentage difference with absolute range of 20%.

Considering the interaction between blood and aortic wall during the cardiac cycle, the maximum displacement of wall reached approximate 0.3 mm in both cases. Taking into account that HOLMES is a representative for both TAWSS and OSI, Fig. 8a–f shows the percentage change in HOLMES in the presence of aortic wall motion. To better comprehend the change, three different color scales were applied on both cases. Figure 8b,e demonstrate that even small amount of wall displacement can cause considerable change of at least $\pm$ 10% in HOLMES and outspreading of over and under estimated values of HOLMES along desired aorta.

## Discussion

Blunt thoracic aortic injury (BTAI) with a timely nature and high mortality rate^2,38,39^, predominantly happens in young adults^39,41^. However, despite promising abilities of computational models along with advances in clinical imaging techniques which is an essence in reinforcement of clinical decisions^25–29^, it seems that the link between clinical researchers of BTAI and invaluable computational haemodynamics is still lacking in the literature.

In this study, a computational model with considering interaction of blood and vessel wall was deployed on a successful TEVAR case for both prior and post intervention. Principal haemodynamic parameters were compared before and after stenting to demonstrate the efficacy of treatment on the patient and also to predict any eventual outcomes after TEVAR. Additionally, in order to study the effect of aortic wall motion, rigid wall simulations were also conducted.

The invasion of the three-layered false lumen created a iatrogenic coarctation segment and the removal of coarcted section of aorta via stenting, led to meaningful enhancement in flow velocity and streamlines boosting downstream perfusion^12^. which finally resulted in increased aortic wall displacement along DA and decreased displacements in upper branches.

Stent deployment resulted in disappearance of disorganised streamlines within the pseudo aneurysm during mid and peak systolic phases. Intra-aneurysmal disordered streamlines are also reported in the study of Tse^42^. Locally narrowed area of the aorta which is located at the end of the pseudo aneurysm can cause helical flows, proximal and distal to the narrowed region specially during deceleration and diastolic phases of cardiac cycle^9–12^; post TEVAR, chaotic swirling flows during these phases do not exist further.

Analysing pressure distribution in computational simulations is vital for predicting possible hypertension. Whether Hypertension is a cause or consequent of stiffening the aortic wall, it can disrupt heart mechanisms in distributing blood to organs and increase the risk of renal complications^43,44^. In current study, results indicated significant pressure decrease during peak systolic phase after TEVAR. This emphasises on efficacy of the treatment which is further validated by the simulation results. However, concerns may rise in younger patients of BTAI who receive an endovascular graft which is much stiffer than young aorta, resulting in aortic wall stiffness and possibility of hypertension^44,45^.

WSS indices are known to be crucial in the study of cardiovascular diseases due to their effect on structure of the vessel wall^46^. Comparing post to pre intervention case, TAWSS had overall reduction which is caused by local uniformity of lumen via stent deployment; specifically, along aortic arch prior to pseudo aneurysm, posterior pseudo aneurysm, and the local narrowing of pre intervention case. This reduction can also be seen in other studies of cardiovascular diseases and will lower the risk of possible future rupture^12,47,48^.

Low magnitude HOLMES areas can be efficacious indicator of atherosclerosis formation and its progression^33^. The stent deployment caused considerable enlargement of areas with low magnitude HOLMES, notably along anterior DA. Thrombus formation for BTAI cases at distal region of the stent graft was confirmed in 26% of the patients after TEVAR^17^ which highlights the importance of regions that are prone for atherosclerosis in vessels and eventually thrombus foundation^50^. Predicting such regions is highly important and requires careful examination during future follow-ups of the patient. Abdoli et al.^51^ also reported a semi-occlusive thrombosis located at distal of the thoracic endo graft nine months after TEVAR for a 29-year-old patient suffering from BTAI which is in agreement with aforementioned statistics and emphasises on the importance of this in silico parameter further.

Comparison of flow irregularities and expressing vortical motions in pre and post intervention cases of the patient, can be done using a vortex identifying parameter which is known as swirling strength (SS)^11,52^. Notable reduction of TASS magnitude via stenting was observed which highlights the effectiveness of TEVAR procedure. The greatest magnitude of SS before intervention, which was seen immediately after the end of pseudo aneurysm (plane eight), was damped, as well as other distal planes after stenting during all three major phases of cardiac cycle. In case of unsuccessful TEVAR, wherein the locally narrowed region still exists, vortical structures with high magnitude of SS gather distal to the coarcted region of the aorta^11^. This eventually leads to destroying the fibrous structure of the aortic wall that may further lead to rupture^11,15^ which is in agreement with high values of Von Mises stress immediately after the local narrowing of pseudoaneurysm prior to intervention. Normalised mean SS in all planes indicated that stent deployment resulted in less vortical strength. However, the magnitude of normalised mean SS is still considerable even after stenting. Some suggest that existence of swirling flow inside the vessels can have clinical benefits as it can suppress flow disturbances caused by stent grafts^53,54^. Based on the same idea helical stent grafts with the ability to induce swirling flow has been proposed that can result in higher WSS and prevent the risk of atherosclerosis^55^; therefore; further study on potentially beneficial range of SS after stenting that can also be tolerated in vessels seems to be needed.

Inclusion of aortic wall motion increases the accuracy of patient-specific simulations theoretically; as neglecting the compliance of vessel wall, may result in neglecting clinical key areas along the aorta^68^. Comparing rigid wall simulations to FSI simulations, significant alter of at least $\pm$ 10% in HOLMES values in the presence of aortic compliance for both pre and post stenting cases was observed. Notable over and underestimated values of HOLMES were outspreaded along the aortae, leading to no significant change of HOLMES distribution pattern in rigid wall simulations compared to FSI simulations^31,56^. What is more, each FSI simulation took more than one week to reach the periodic steady-state due to the excessive computational cost of FSI simulations^31,56–58^. This cost is not practical in BTAI cases due to emergent nature of BTAI^2^; therefore, compromising the motion of the vessel wall in future studies can be considered to obtain a more clinical-friendly patient-specific CFD model^56^.

To the best of author’s knowledge, the present work was the first attempt to propose an FSI model to study the change of key haemodynamic parameters in a clinical case of BTAI after successful TEVAR. The 3D patient-specific geometries of the patient were coupled with three-element Windkessel model in a two-way fluid–solid interaction study, wherein blood was considered a non-Newtonian fluid in both pre and post intervention cases. However, compromising the inclusion of aortic wall motion in future studies which include comparison of patient’s condition before and after treatment may be considered due to excessive computational costs and should be based on the objectives of the study. Results showed significant enhancement in velocity and pressure distribution after stenting. Since BTAI studies have reported thrombus formation at the distal aspect of the stent graft, areas with low values of HOLMES after intervention require careful examination in future follow-ups. In addition, SS along aorta was also damped after stent deployment, requiring a further investigation for a clinical-friendly range of SS in patients that are TEVAR candidates.

## Methods

To deploy the computational model, a successful TEVAR case was chosen and the study was performed under IR.IUMS.FMD.REC.1401.257 ethic number. The study protocol was approved by the Rajaie Cardiovascular Medical and Research Center ethics committee and the participant signed informed consent and all methods were performed in accordance with the relevant guidelines and regulations. Patient investigated herein is a 30-year-old male who had been admitted to emergency department of Rajaie Cardiovascular Medical and Research Center as a result of motor falling down from four-meter height with no known past medical history of any cardiovascular disease. Apart from concomitant skull, T11–12 spine and femoral fractures, initial CTA revealed a pseudo aneurysmal formation beyond the left subclavian artery consisted with blunt aortic injury. TEVAR was deemed necessary and proceeded as emergent basis. A 26 × 105 mm Zenith Thoracic Endovascular graft (Cook Medical, Bloomington, Ind) was deployed from the left femoral artery with acceptable immediate result. One, six and twelve-month follow-up CTA revealed no complication.

### Patient-specific geometry

**Figure 9:**
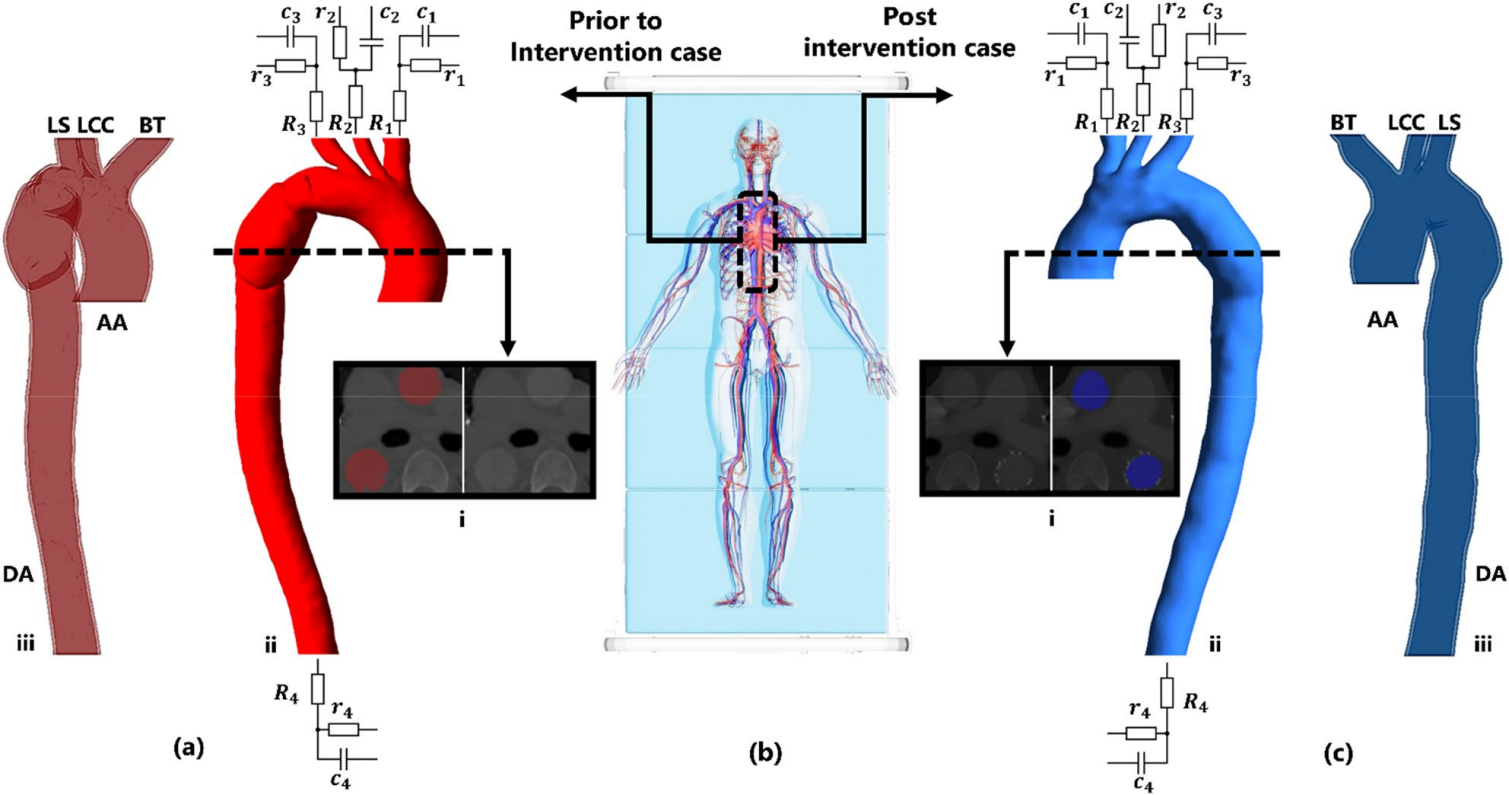
Patient-specific domains and boundary conditions. (**a.i**) and (**c.i**) CT scan images of prior and post intervention cases, (**a.ii**) and (**c.ii**) Fluid domains and 3-Element Windkessel model for both cases, (**a.iii**) and (**c.iii**) Solid domains, and (**b**) Desired region.

The three dimensional (3D) fluid domain (blood) of a patient suffering from aortic blunt trauma was extracted. The affected region starts immediately after the arch and it ends prior to distal abdominal as shown in Fig. 9b. For the purpose of both capturing the affected region on upstream or downstream of blunt trauma region and boosting computational time, further downstream regions were not captured for this study. The approximate length of the 3D domain was 200 mm; including some regions of brachiocephalic trunk, left common carotid, left subclavian arteries, and distal abdominal, prior to the renal arteries and the iliac bifurcation.

Two sets of CT images were available with 1723 and 376 digital imaging and communications in medicine (DICOM) images and slice interval of 0.5 mm and 1 mm for prior and post invasive intervention respectively.

DICOM images for both prior and post intervention were imported into MIMICS Research 21.0 (Materialise, Leuven, Belgium) wherein the region of interest for both domains were extracted. To separate the region of interest (aorta) from the rest of organs and vessels, several masks and tools were applied. Figure 9a.i, c.i show the final reconstructing 3D fluid domains. The 3D fluid domains were wrapped and smoothed to improve each pixel evenness and to ease the process of discretising in further stages of preparing the computational domains.

Both fluid domains were imported into CATIA V21 (Dassault Systems, Velizy-Villacoublay, France) wherein the boundaries where cropped perpendicular to the z-axis. The final models are shown in Fig. 9.a.ii,c.ii for prior and post intervention respectively. Blood enters the ascending aorta (AA) and flows through the upper branches, aortic arch and through the narrowed region toward distal abdominal (DA).

Standard trauma protocol at Rajaie Cardiovascular Medical and Research Center limits the resolution and slice interval of CT images; as a result, it was not possible to capture wall of the aorta for this study. In a previous study of Aortic Blunt Trauma, a simple model of aortic wall with constant thickness along aorta was used^24^. Furthermore, it has been reported that although aortic wall thickness varies significantly in the population^59^, values of less than 4 mm is considered normal^60^, thus 1.5 mm thickness was chosen.

To generate the aortic wall, 3D fluid domains were imported into SpaceClaim 2019 R3 (ANSYS Inc., Canonsburg, PA, USA). Faces of fluid domains were fixed and extruded by 1.5 mm normal to every face. The generated solid domains were cut with respect to fluid domains as shown in Fig. 9a.iii,c.iii for both cases.

### Discretising domains (meshing)

For the purpose of computational investigation, both fluid (blood) and solid (vessel) domains were discretised into small elements wherein crucial haemodynamic parameters were further investigated. The fluid and solid domains were meshed separately using ANSYS Workbench 2019 R3 (ANSYS Inc., Canonsburg, PA, USA). Both fluid and solid were discretised with tetrahedral elements and to create high quality geometry-aligned elements near the aortic wall, five prismatic layers were used at the wall of the fluid domains. For the purpose of sensitivity analysis, three sets of mesh elements were used for all cases and eventually to compromise for both accuracy and computational time, the medium mesh with approximate 300,000 and 40,000 elements were chosen for fluid and solid domains respectively.

### Boundary conditions (BC)

Due to the emergent nature of these trauma and the need for immediate intervention, patient-specific inlet velocity data prior to intervention was not available. In addition, for the purpose of consistency an inlet velocity profile was deployed for both prior and post ascending aortic velocity^61^. Thus, the inlet velocity profile of the patient was calculated based on an ideal half sine signal taken from Ventre et al.^62^.

5 $$V\left(t\right)=\left\{\begin{array}{ll}{V}_{0}\mathit{sin}\left(\frac{\pi t}{{T}_{ej}T}\right)&\quad if \,0<t<{T}_{ej}T\\ 0&\quad if\, {T}_{ej}T\le t<T\end{array}\right\}$$

where ${V}_{0}$ is the maximum velocity of blood at inlet, ${T}_{ej}$ is the systolic ejection time and $T$ is the length of cardiac cycle. Using the post-intervention pulsed wave Doppler echocardiography (PW Doppler ECHO) image of the patient, ${V}_{0}$ was reported to be $96.1\frac{\mathrm{cm}}{\mathrm{s}}$, and a ${T}_{ej}$ of $0.273 \mathrm{s}$ can be achieved. What is more, the length of cardiac cycle was $0.731 \mathrm{s}$ taking into consideration the heartbeat per minute (BPM) of the patient for post intervention.

The outlet boundaries of both prior and post intervention fluid domains were coupled with three element Windkessel models. To calculate Windkessel parameters, the systolic and diastolic pressure for each outlet were needed. The systolic and diastolic blood pressure for this patient were set to 120 mmHg^63^ and 77.5 mmHg^32^ respectively. Process of deriving and tuning Windkessel parameters for each outlet was based on the work of Alimohammadi^65^. Final tuned Windkessel parameters are listed in Table 2.

**Table 2:** Final tuned Windkessel parameters.

	BT	LCC	LS	DA
R (mmHg s/ml)	9.98	47.62	46.01	1.03
r (mmHg s/ml)	0.03	0.50	0.12	0.16
C (ml/mmHg)	0.41	0.53	0.38	1.36

Blood was considered to be an incompressible non-Newtonian fluid with density of 1056 kg/m^3^ for both prior and post intervention^31^. To exhibit the non-Newtonian behavior of blood, Carreau–Yasuda model was used. According to:

6 $$\mu =\left({\mu }_{0}-{\mu }_{\infty }\right){\left(1+{\left({\lambda }_{CY}{\gamma }^{\mathrm{^{\prime}}}\right)}^{a}\right)}^{(m-1)/a}+{\mu }_{\infty }$$

wherein $\mu$ is viscosity, ${\mu }_{0}$, ${\mu }_{\infty }$, ${\lambda }_{CY}$, $\gamma \mathrm{^{\prime}}$, $a$, and $m$ are low shear viscosity, high shear viscosity, time constant, shear rate, Yasuda exponent, and power law index respectively, $\mu$ is a function of five parameters^66^ that are presented in Table 3.

**Table 3:** Parameters of Carreau-Yasuda blood viscosity model^66^ and Material constants of aortic wall^67^.

Carreau–Yasuda constants					Hyperelasticity constants	
${\mu }_{0}$(Pa s)	${\mu }_{\infty }$(Pa s)	${\lambda }_{CY}$(s)	$a$	$m$	A (MPa)	B (MPa)
0.0220	0.0022	0.1100	0.6440	0.3920	0.1740	1.8810

To avoid under-predicting swirling effects of blood flow on aorta, a Shear–Stress Transport (SST) model with intensity of 1% was used for both cases. Hyperelastic behavior of aortic walls were modelled using a two-parameter Mooney-Rivlin model^67^. This model better adopts the mechanical properties of the vessel wall and provides computational robustness. The material constants of this model are presented in Table 2.

### Computational fluid dynamics (CFD)

To consider the interaction of blood and aortic wall, a two-way fluid–solid interaction (FSI) model was used for both prior and post intervention. The properties and BCs of fluid and solid domains were implemented through CFX 2019 R3 (ANSYS Inc., Canonsburg, PA, USA) and Transient Structural (ANSYS Inc., Canonsburg, PA, USA), respectively. The interface of solid domain with fluid domain was initialised within the range of diastolic pressure of the patient and to compensate the elasticity of aortic wall layers a number of springs were coupled with inner and outer surfaces of aortic walls for both cases. The continuity and Navier–Stokes equations of fluid domain were coupled with the motion of solid domain using ANSYS System Coupling 2019 R3 (ANSYS Inc., Canonsburg, PA, USA) for both cases.

For rigid cases simulations were conducted on fluid domains with the same BCs applied for FSI models, neglecting the motion of solid domain. These simulations are so called “rigid wall “simulations and were carried out for the purpose of comparison^68^.

In all simulations the time step was set to $0.002 \mathrm{s}$ and residual mean square errors were held below $1\times {10}^{-5}$.

### Limitations

In this study improvement of haemodynamic parameters were achieved only through the change of geometry after stenting. However, the effect of pharmaceutical treatments (beta-blockers) and diet were not taken into account. In addition, in the aforementioned patient, stent deployment revealed no complication after TEVAR in terms of expanding the lumen. Therefore, thickness and elasticity of the stent have been compromised herein to reduce complexity of the current model. What is more, although the characteristics of wall change after TEVAR, in current study material properties and thickness of aortic wall for both prior and post intervention cases were assumed to be the same. More realistic simulations can be conducted in case of availability of data on aortic wall thickness before and after treatment of BTAI.

Coarcted regions of aorta and their down streams are known to be at risk of failure and therefore rupture. To the best of our knowledge no experimental data for these regions regarding elasticity and Poisson's ratio is available. Therefore, to avoid adding extra supports along these regions and to compensate the elasticity of aortic wall layers a number of springs were coupled with inner and outer surfaces which resulted in less displacement in comparison with magnitudes reported in the literature. More realistic FSI models could be proposed in case of data availability.

## Data Availability

The datasets used during the current study is available from the corresponding author on reasonable request.
