# Author Correction: In-silico investigations of haemodynamic parameters for a blunt thoracic aortic injury case

**DOI:** 10.1038/s41598-023-37106-z

**Published:** 2023-06-20

**Authors:** Rezvan Dadras, Alireza Jabbari, Narges Kamaei Asl, Madjid Soltani, Farnaz Rafiee, Mozhgan Parsaee, Shadi Golchin, Hamidreza Pouraliakbar, Parham Sadeghipour, Mona Alimohammadi

**Affiliations:** 1 Department of Mechanical Engineering, K. N. Toosi Univeristy of Technology, Tehran, Iran; 2 Rajaie Cardiovascular, Medical, and Research Center, Iran University of Medical Sciences, Tehran, Iran

Correction to: *Scientific Reports* https://doi.org/10.1038/s41598-023-35585-8, published online 23 May 2023.

In the original version of this Article, Rezvan Dadras was incorrectly listed as a corresponding author. The correct corresponding author for this Article is Parham Sadeghipour, as well as Mona Alimohammadi. Correspondence and request for materials should be addressed to psadeghipour@hotmail.com or mona@alimohammadi.co.uk.

The original Article has been corrected.

